# Promising Health Benefits of Adjuvant *Acmella* and *Zingiber* Extracts Combined with Coenzyme Q10 Phytosomes, Supplementation in Chronic Pain Treated with Medical Cannabis: A Prospective and Open-Label Clinical Study

**DOI:** 10.1155/2022/7099161

**Published:** 2022-06-13

**Authors:** Paolo Poli, Simona Carnevale, Antonella Scocca, Pier Luigi Davolio, Simona Busi, Martino Meneghin, Giovanna Petrangolini, Antonella Riva

**Affiliations:** ^1^Polipain Clinic, Pisa, Italy; ^2^Farmad Lab-R & D, Florence, Italy; ^3^Indena SpA, Research and Development Department, Milan, Italy

## Abstract

**Background:**

Chronic pain is a condition where pain persists for months or even years. Nowadays, several drugs comprising of medical cannabis are utilized for chronic pain relief. Even if there are some associated side effects, the use of supplements can widen the reliable tools available for improving an individual's quality of life.

**Objective:**

The aim of the present study was to evaluate the efficacy in terms of pain intensity, psychological well-being, and quality of life of a new dietary supplement in chronic pain subjects under current treatment with medical cannabis.

**Methods:**

In this pilot study, 48 medical cannabis-treated subjects were supplemented with a dietary supplement containing a combination of standardized *Zingiber officinalis* and *Acmella oleracea* extracts in phytosome (Mitidol), coenzyme Q10 phytosome (Ubiqsome), and group B vitamins (B1, B6, and B12), twice daily for 90 days. In order to explore the benefits of the product as an adjuvant supplementation for pain relief, the pain intensity, measured by the visual analogue scale (VAS), the pain type, and quality, evaluated by the Italian Pain Questionnaire (QUID) and the possible reduction of therapeutic and/or painkiller doses were recorded.

**Results:**

After 90 days, significant pain relief was detected in almost 70% of the subjects receiving the new dietary supplement, with sensory, emotional, and pain amelioration in one-third of them. A reduction in both tetrahydrocannabinol (THC) and cannabidiol (CBD) doses was also observed after 3 months of supplementation. These findings demonstrate new perspectives for the use of an innovative dietary supplement that combines *Acmella* and *Zingiber* extracts, Coenzyme Q10, and group B vitamins resulting in a beneficial long-term adjuvant in cannabis-treated pain subjects.

## 1. Introduction

Chronic pain is generally defined as pain refractory to standard treatment modalities. It is a stressing condition affecting 100 million people in Europe, impacting subjects' quality of life. Different types of chronic pain exist, with the most represented being neuropathic pain, rheumatoid arthritis, headache, fibromyalgia, and central nervous system (CNS) disorders [1].

Cannabinoid prescriptions in Italy [2–5] are allowed for chronic pain and pain associated to multiple sclerosis, as well as other indications (i.e., HIV and cancer) [6–8]. However, therapy with medical cannabis would also be insufficient to control chronic pain or would cause collateral effects such as sleep problems, anxiety, or stress [9, 10]. Supplementation with natural products could help to reduce cannabis-related side effects or cannabis/painkiller use.

Therefore, the aim of the present investigation was to observe potential benefits of a long-term administration (90 days) of a dietary supplement containing both Mitidol and Ubiqsome phytosomes with group B vitamins to subjects with chronic pain under treatment with medical cannabis.

Previously published data already demonstrate that each phytosome has anti-inflammatory, antioxidant, and pain relief properties [11, 12] that are probably also linked to CB2 modulation [13].

An improvement would be expected in general well-being and ache positive modulation in chronic pain where the endocannabinoids system plays an important role.

## 2. Methods

### 2.1. Study Design

The study was an open pilot study of 12 weeks (90 days) with adjuvant supplementation, carried out in 48 subjects (mean ± SD age 58.56 ± 16.6 years) including 10 males (20.83%) and 38 females (79.17%). Different types of chronic pain were considered as detailed hereafter: 4 subjects (8.3%) with headache and migraine, 9 subjects (18.7%) with CNS disorders, 26 subjects (54.2%) with rheumatic autoimmune diseases, and 9 subjects (18.7%) with neuropathic pain.

#### 2.1.1. Inclusion Criteria

Inclusion criteria of the study were chronic pain, use of traditional painkillers for at least three months without satisfied efficacy, capacity to understand the research process fully and consciously, and sufficient ability to provide informed consent.

#### 2.1.2. Exclusion Criteria

Exclusion criteria were organic or psychiatric disorders interfering with judgment and understanding, pain for less than three months, allergies to the ingredients contained in the food supplement, pregnancy, lactation, or presence of arrhythmias or ischemic heart disease.

#### 2.1.3. Main Variables

The main variables assessed during the study were as follows:

  Pain intensity, evaluated by the following tools: clinical characteristics of pain (Italian Pain Questionnaire, QUID) [14];
  Hospital Anxiety and Depression Scale (HADS) [15], a self-assessment scale developed to detect depression, anxiety, and distress in subjects with chronic diseases, which discriminated between psychopathological or somatic conditions and included 14 items: seven for anxiety and seven for depression;
  Visual Analogue Scale (VAS), which allowed subjects to evaluate their pain level between 0 (“no pain”) and 10 (“worst possible pain”)
  Presence/absence of a range of benefits (pain relief/suppression, muscle relaxation, mental relaxation, increased activation, improved sleep quality, and general quality of life recovery)
  Presence/absence of a range of side effects (poor concentration, mental confusion/disorientation; tachycardia, dry mouth, drowsiness, multiple side effects, increased appetite, and hyperactivity/restlessness)
  Reduction of the use of medical cannabis dose and/or painkiller drugs

The investigated variables were measured at baseline (time 0) and every 10 days for three months (time 9) to highlight correlations and potential changes in time. Enrollment was conducted at the medical pain therapy clinic Poli Pain Clinic s.r.l., Via Ippolito Nievo 23, Cascina (Pisa, Italy).

All the procedures complied with the ethical standards of the committee in charge of human study evaluation. The study was carried out in compliance with and according to the basic clinical principles, the operational principles, and additional guidelines of the sixth and last 2008 version of the Helsinki Declaration. A notification to the Ethical Committee of Azienda Toscana Sud-Est, Arezzo has been successfully applied, and the study was approved by the ethics committee (no. 1961 05/15/2018).

Upon enrollment, each subject was asked to express his or her consent to participate in writing, after receiving the due and necessary explanations and a detailed and exhaustive description of the study. Each subject could withdraw his or her consent to participate in the study at all times.

The personal data were processed in view of maximum privacy protection, according to the legal provisions in force, and subjects' anonymity was carefully protected.

### 2.2. Supplement

All the subjects were under treatment with medical cannabis, a combination of CBD and THC from 0.02 to 2 mg to about 20 mg, depending on the subject's clinical status, according to the Italian requirements for pharmaceutical preparations (as reported by the Italian Society of Pharmacists [16, 17]), for chronic pain. The dietary supplement was administered as 2 tablets/day (one tablet in the morning and one tablet in the evening) for 90 days.

The dietary supplement consisted of Mitidol and Ubiqsome, formulated in tablet form (ZAC Q10, Farmad Laboratori Firenze s.r.l., Italy). In detail, each tablet contained the *Zingiber officinale* and *Acmella oleracea* standardized extracts formulated with the phytosome technique [11] (Mitidol™, Indena S.p.A.) and coenzyme Q10 formulated as a phytosome [18] (Ubiqsome™, Indena S.p.A.), together with B1, B6, and B12 vitamins.

The amount of the single components in each tablet was the following: 350 mg Mitidol, 150 mg Ubiqsome, 0.55 mg thiamine hydrochloride (B1), 0.7 mg pyridoxine hydrochloride (B6), and 1.25 *μ*g cyanocobalamin (B12).

### 2.3. Data Management

Data were safely managed and stored, and the name of each subject was anonymized via the automatic generation of a code produced according to the origin and progressive number.

Statistical analysis was performed by analysis of variance (ANOVA) one-way for repeated measures (for normal distribution) or the Friedman test followed by Dunn's test for multiple comparisons (for nonnormal distribution). Statistical analysis between T0 and T9 mean values ± S.D. was performed by the Wilcoxon matched-pairs signed rank test. Linear regression analysis was also performed.

## 3. Results

**Figure 1:**
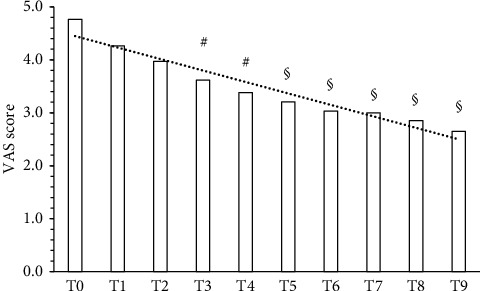
Pain relief during supplementation up to 90 days in subjects (70.83%, excluded dropouts) affected by chronic pain of different etiologies. Data are analysed by the Friedman test: *F* (9.297) = 162.10, *p* < 0.0001; Dunn's test: ^#^*p* < 0.01 and ^§^*p* < 0.0001 vs. T0 (baseline). Linear regression analysis: *r*^2^ 0.9319 (*p* < 0.0001).

**Figure 2:**
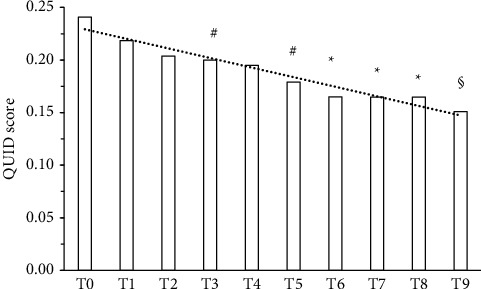
QUID TOT up to 90 days supplementation in subjects (70.83%, excluded dropouts) affected by chronic pain of different etiology. Data are analysed by the Friedman test: *F* (9.297) = 70.23, *p* < 0.0001; Dunn's test: ^#^*p* < 0.05, ^*∗*^*p* < 0.001, and ^§^*p* < 0.0001 vs. T0 (baseline). Linear regression analysis: *r*^2^ 0.9506 (*p* < 0.0001).

**Figure 3:**
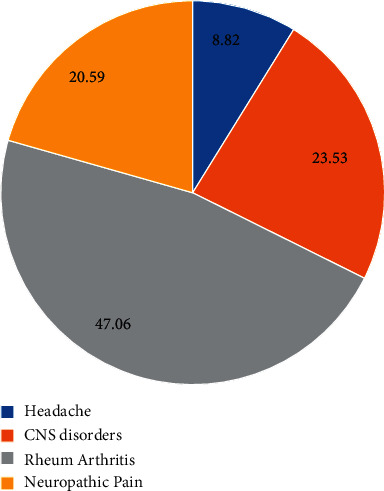
Chronic pain types (%) in responsive subjects. Data are expressed as % responsive subjects.

A statistically significant reduction (*p* < 0.0001) in pain as VAS mean was detected after 3 months supplementation in 34/48 subjects (70.8%) (Table 1 and Figure 1). QUID TOT mean values were also statistically significantly reduced after 90 days supplementation (Table 1 and Figure 2). One-third of the responsive subjects showed a complete benefit profile (sensory, emotional, and pain), while the remaining part displayed one or two benefits. In terms of improvement, while 20% reported only pain relief, 8.8% also displayed emotional improvement. A significant positive linear correlation among supplement and reduction of both VAS and QUID TOT with the time was also observed (Figures 1 and 2). In the responsive subjects group, those having rheumatoid arthritis were the most abundant (47.06%), followed by subjects with CNS disorders (23.53%), neuropathic pain (20.59%), and headache (8.82%) (Figure 3).

During the 3 months supplementation, a change in the medical cannabis dosage was applied in 16/34 subjects. Among them, 9 subjects (56.25%) had their medical cannabis dose reduced, scaling down on average by 2.40 mg/day of THC and 0.94 mg/day of CBD; two of these subjects also withdrew from the use of painkiller drugs. For one subject, the THC dose was reduced, but the CBD amount was increased, while another subject was treated with an increased THC dose and a smaller CBD dose. On the contrary, five subjects needed a dose increase for both active ingredients of medical cannabis by 3.24 mg/day of THC and 1.19 mg/day of CBD on average; two of the five subjects withdrew from painkillers. In summary, at the end of the study, an average dose reduction of 0.31 mg/day for THC and 0.14 mg/day for CBD was registered.

A dropout of 12 subjects (25%) was recorded: four subjects for lack of benefits and onset of side effects, such as abdominal pain and gastric reflux, one subject for significant gastric reflux, despite reporting pain relief, three subjects for lack of pain-related benefits, three subjects for poor compliance, and one subject at the beginning of month three for a worsening of his clinical conditions due to SARS-CoV-2 infection.

## 4. Discussion

In the present study and for the first time, a new dietary supplement combining Mitidol and Ubiqsome (ZAC Q10) showed that a long-term supplementation with *Acmella* and *Zingiber* extracts, coenzyme Q10 phytosome, and group B vitamins resulted in a beneficial health amelioration for the relief from pain in medical cannabis-treated subjects.

Chronic pain subjects experience continuous recurring pain at monthly or yearly intervals, which makes it impossible to carry out ordinary daily activities [19]. Current therapies for chronic pain included nonsteroidal anti-inflammatory drugs, opioids, antidepressants, and myorelaxant agents. However, due to side effects and a low efficacy, the pain intensity remains of considerable clinical importance. The discovery of the crucial role of the endocannabinoid system in pain opened new therapeutic perspectives [20, 21], and the introduction of cannabis therapy for chronic pain has been successful [3, 22], as demonstrated by several studies [23–26]. Medical cannabis products were recently well-summarized by Brunetti et al. [5], with the rationale to help doctors with dosing and titration strategies for THC and CBD preparations.

Therefore, an adequate formulation of oral cannabis was also fundamental for the partial relief from particular severe chronic pain [4, 27].

Considering that medical cannabis therapy of chronic pain is often associated with side effects [9, 10], we explored the potential ability of a daily coadministration with a dietary supplement for 3 months, specifically developed for the mitigation of pain through healthy inflammation support and endocannabinoid system modulation.

Previous studies support the activities of *Acmella oleracea* and *Zingiber officinalis* extracts formulated in phytosomes as endocannabinoid modulators and natural adjuvants in pain management [11, 13, 28].

In the present study, this ingredient appeared to synergize with medical cannabis treatment in 53% of responsive subjects, leading to a reduction of the therapeutic daily cannabis dose together with pain relief. Only one subject that used high doses of THC reported complaints during treatment, which can be attributed to a more powerful effect of THC.

Interestingly, among the 15 subjects that did not perceive any benefit during the first month, 9 reported a significant pain reduction during the second and third month.

The results obtained showed that a 3-month supplementation with this new dietary supplement allowed important benefits on pain perception and emotional feeling, maintaining a good safety profile.

The presence of a bioavailable CoQ10 formulated as a phytosome [18] would be helpful due to its ability to reduce oxidative stress-related chronic pain, most likely due to its antioxidant and anti-inflammatory properties [29].

Interestingly, subjects with chronic inflammatory pathologies like rheumatoid arthritis benefited from the adjuvant supplement. The results are in agreement with a clinical study recently published [11], where the food-grade lecithin formulation of standardized extracts of *Zingiber officinale* and *Acmella oleracea* showed a statistically significant effect on reducing symptoms of knee osteoarthritis (OA) (pain and knee function), with anti-inflammatory activity demonstrated by a reduction of specific biomarkers (C-reactive protein (CRP) and erythrocyte sedimentation rate (ESR)).

Of note, the positive health benefits of the dietary supplement were observed not only in arthritis pain but also in different physiological contexts, underlining the cross-sectional potential of the product.

Due to the pilot design of this study, some limitations are present. The small sample size of the recruited population was determined by the feasibility of enrollment. The second limitation might be represented by the lack of both randomization and controlled groups. Those features are typical of a feasibility investigation that could be extended with a trial performed with a control group and in a randomized way. However, the results obtained in the present study confirm the safety and beneficial activity of the adjuvant supplement on the endocannabinoid system and its potential use for chronic pain of different etiologies.

## 5. Conclusion

In conclusion, the use of *Zingiber officinalis* and *Acmella oleracea* extracts together with CoQ10 in phytosomes resulted in safe and promising long-term pain relief, with a reduction in medical cannabis dosages and painkillers use. Further clinical studies, possibly randomized, double-blind, controlled ones, with a larger number of subjects, are still advised.

## Figures and Tables

**Table 1:** Baseline characteristics and main positive effects of supplementation in subjects (70.83%, excluded dropouts) affected by chronic pain of different etiologies.

Subjects	Before supplementation, T0	After 90 days supplementation, T9	Statistical analysis vs. T0
Number	34	34	
Sex (male/female)	7/27	7/27	
VAS (mean ± S.D.)	4.8 ± 1.9	2.6 ± 1.9	*P* < 0.0001
QUID TOT (mean ± S.D.)	0.24 ± 0.16	0.15 ± 0.14	*P* < 0.0001
Benefits	—	Sensory, emotional, pain (35.3%)	
	—	Sensory, emotional (5.9%)	
	—	Sensory (14.7%)	
Improvements	—	Pain evaluation, emotional (2.9%)	
	—	Emotional (8.8%)	
	—	Pain (5.9%)	

Results are expressed as mean ± standard deviation (S.D.); —, not applicable. VAS : visual scale assignment; QUID: Italian pain questionnaire. Mean data analysis, Wilcoxon matched-pairs signed rank test.

## Data Availability

The data used to support the findings of this study are available from the corresponding author upon request.
